# SATRAT: *Staphylococcus aureus* transcript regulatory network analysis tool

**DOI:** 10.7717/peerj.717

**Published:** 2015-01-06

**Authors:** Tamilselvi Gopal, Vijayaraj Nagarajan, Mohamed O. Elasri

**Affiliations:** 1 Informatics LLC, Silver Spring, MD, USA; 2 Bioinformatics and Computational Biosciences Branch (BCBB), Office of Cyber Infrastructure and Computational Biology (OCICB), National Institute of Allergy and Infectious Disease (NIAID), National Institutes of Health (NIH), Bethesda, MD, USA; 3 Department of Biological Sciences, The University of Southern Mississippi, Hattiesburg, MS, USA

**Keywords:** Transcriptome, RNA sequencing, *Staphylococcus aureus* transcriptome meta-database (SATMD), RNA-Seq, *Staphylococcus aureus* microarray meta-database (SAMMD), *Staphylococcus aureus*, Transcript regulatory network, Infectious disease

## Abstract

*Staphylococcus aureus* is a commensal organism that primarily colonizes the nose of healthy individuals. *S. aureus* causes a spectrum of infections that range from skin and soft-tissue infections to fatal invasive diseases. *S. aureus* uses a large number of virulence factors that are regulated in a coordinated fashion. The complex regulatory mechanisms have been investigated in numerous high-throughput experiments. Access to this data is critical to studying this pathogen. Previously, we developed a compilation of microarray experimental data to enable researchers to search, browse, compare, and contrast transcript profiles. We have substantially updated this database and have built a novel exploratory tool—SATRAT—the *S. aureus* transcript regulatory network analysis tool, based on the updated database. This tool is capable of performing deep searches using a query and generating an interactive regulatory network based on associations among the regulators of any query gene. We believe this integrated regulatory network analysis tool would help researchers explore the missing links and identify novel pathways that regulate virulence in *S. aureus*. Also, the data model and the network generation code used to build this resource is open sourced, enabling researchers to build similar resources for other bacterial systems.

## Introduction

*Staphylococcus aureus* is a commensal organism that primarily colonizes the nose of healthy individuals. The close association of *S. aureus* with the host provides an ideal setting to initiate opportunistic infections. *S. aureus* causes a spectrum of infections that range from skin and soft-tissue infections to fatal invasive diseases. Additionally, the emergence of methicillin-resistant *S. aureus* (MRSA) strains has complicated the control of staphylococcal infections because these strains are resistant to all *β*-lactam antibiotics, which have traditionally been used in therapy. Outbreaks of MRSA strains were initially associated with hospital settings where they caused up to 64% of staphylococcal infections in intensive care units (Klevens et al., 2006). The last two decades have witnessed a disturbingly rapid emergence of MRSA infections outside the healthcare setting and in the community (Chambers, 2001). The pandemic of these community-associated MRSAs (CA-MRSA) is caused by only a few clones (e.g., USA300) that are highly virulent and are sustained in the population by rapid spread (Francis et al., 2005; Miller et al., 2005; Diep & Otto, 2008; Hidron et al., 2009).

The success of *S. aureus* pathogenicity is due to the large number of virulence factors it produces, its adaptability to various environments (e.g., host), and the presence of nutrients or stressors. *S. aureus* has a very intricate network of regulators that allows *S. aureus* to survive or thrive in various environments. Indeed, *S. aureus* encodes 135 transcription factors and sigma factors (Ibarra et al., 2013). Several genes in *S. aureus* are considered to be global regulators because they control the expression of numerous genes (Nagarajan, Smeltzer & Elasri, 2009). Transcriptomics have been used as a powerful tool to study this pathogen. Analysis of the transcriptome in a cell helps to understand the function of each gene in the context of a whole system. Microarray and next-generation sequencing of transcripts (RNA-Seq) are the two commonly used methods to analyze transcriptomes and investigate cellular state, activity, and physiology. The *S. aureus* microarray meta-database (SAMMD) (Nagarajan & Elasri, 2007) was the first of its kind to collect, curate, compile, and develop a user-friendly interface of all the published transcriptome data of *S. aureus*.

In this paper, we describe the *S. aureus* transcript regulatory network analysis tool (SATRAT), which is based on the *S. aureus* transcriptome meta-database (SATMD)—a substantially updated version of the previously published SAMMD (Nagarajan & Elasri, 2007). We believe SATRAT will allow researchers to understand and discover hidden links between the numerous regulatory elements of *S. aureus*.

## Materials and Methods

### Updates to the database

SATMD is the significantly updated version of SAMMD. The schema of SAMTD contains tables describing the lists of regulated genes, experiment details, annotations, and references to publications. SATMD has an additional data column in the experiments table that classifies the transcriptome technology that was used to generate the data (e.g., RNA-Seq or microarray).

Newly published papers in PubMed describing transcriptome experiments were identified using the search terms stimulon, transcriptome, transcriptomics, transcription profile, transcription profiling, and microarray in combination with *Staphylococcus aureus*. Data extraction and curation were done as previously described (Nagarajan & Elasri, 2007). In summary, the extracted lists of differentially expressed genes were mapped to *S. aureus* strain N315 IDs, and redundancies were removed. Relevant experimental details were extracted by careful reading of the published articles. The quality of the data was checked using in-house perl scripts that identify any discrepancy between the number of extracted IDs and mapped IDs.

**Figure 1:**
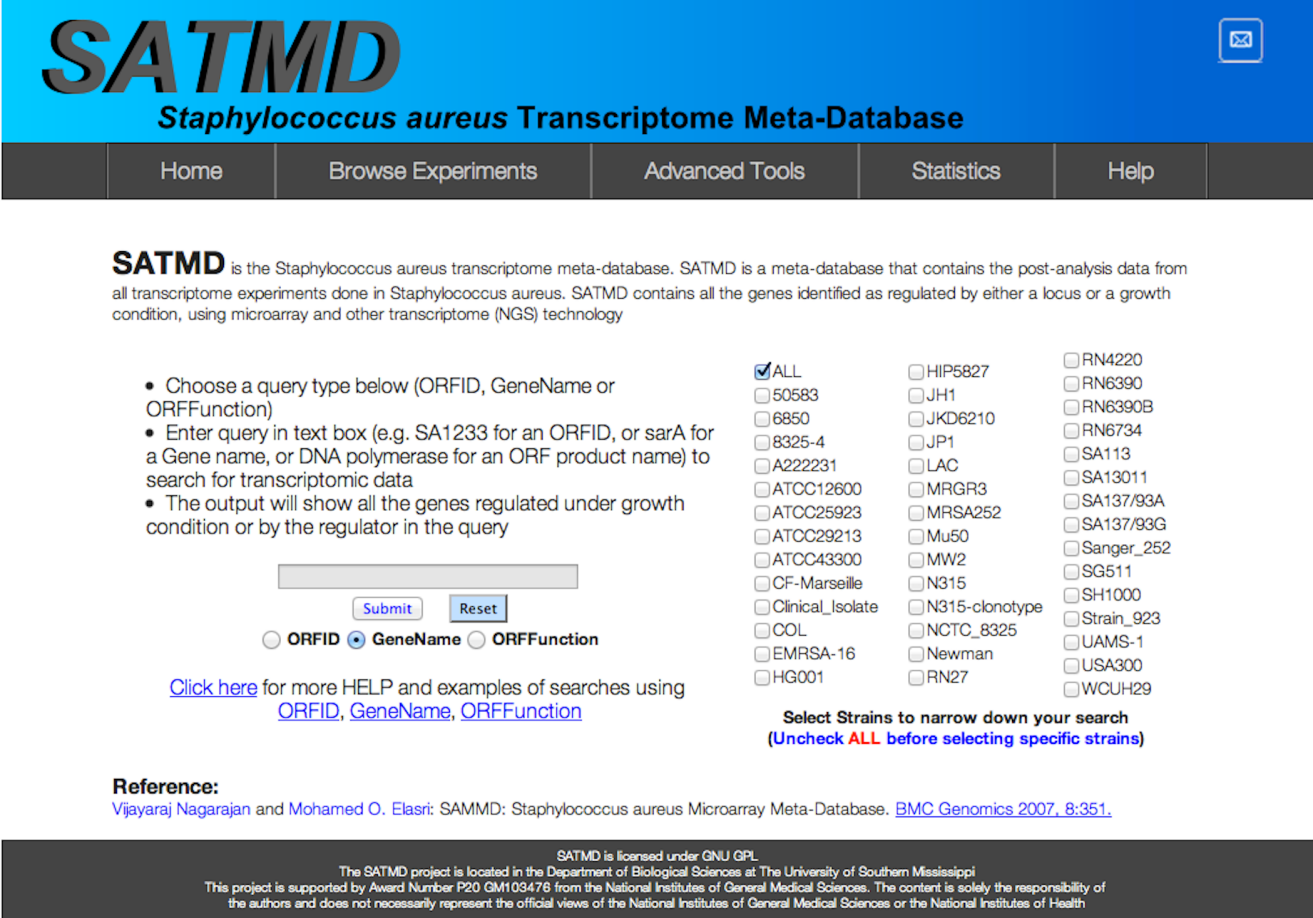
Redesigned SATMD. Redesigned *Staphylococcus aureus* transcriptome meta-database (SATMD) website home page showing 42 strain-based search filters.

While most of the core search, filter, and browsing features are retained, the user interface has been redesigned (Fig. 1: Redesigned SATMD website home page, with 42 strain-based search filters) using the latest HTML5 standards to accommodate modern browsers and computer displays.

An updated ID mapping file has been created to include four new genome sequences (NEWMAN, USA300, NCTC8325, and USA300TCH1516) in addition to the previously available ones (MW2, Mu50, COL, N315, MRSA252, and MSSA476). SATMD also includes added search filters for 22 new strains in addition to the 20 old strain filters. SATMD currently contains data from 250 experiments including experiments for 92 gene-based transcriptomes and 158 experimental condition-based transcriptomes. SATMD contains data extracted from 112 peer-reviewed publications that span the period from 2001 to 2014 and is continually updated.

### *S. aureus* transcript regulatory network analysis tool (SATRAT)

**Figure 2:**
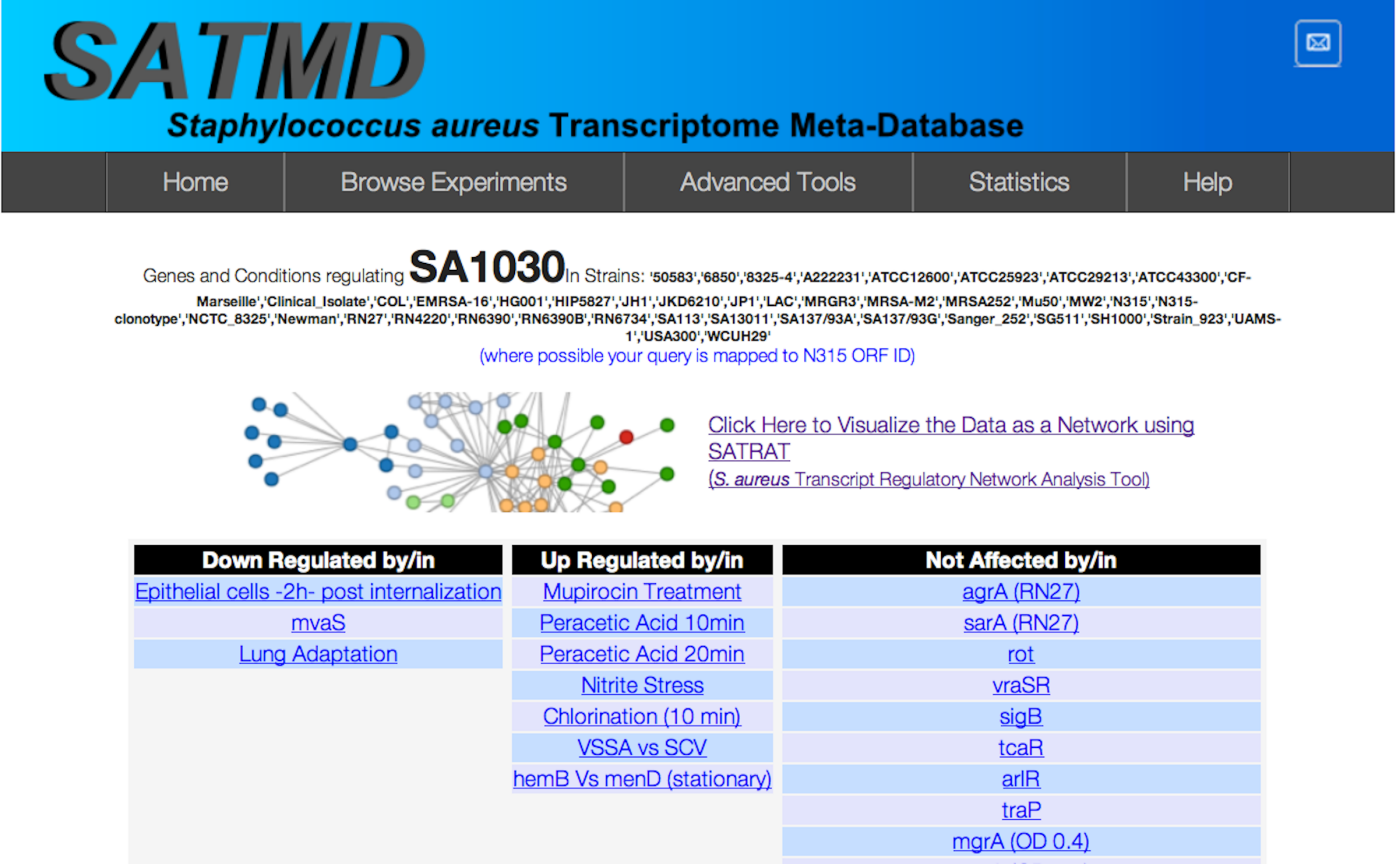
Search result. SATMD basic gene search result for the hypothetical protein SA1030.

**Figure 3:**
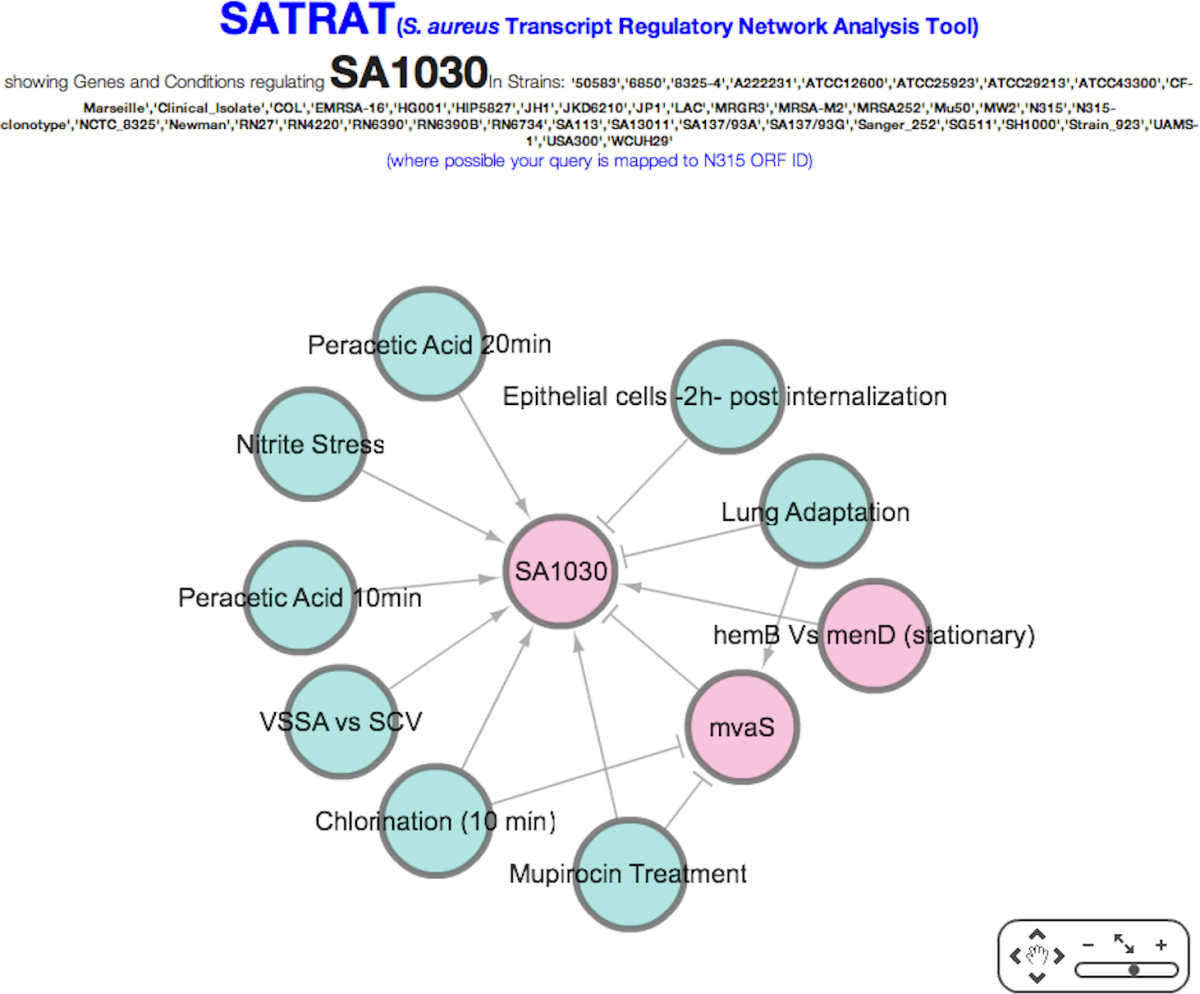
SATRAT. Visualization of associations among regulators of SA1030 using the *Staphylococcus aureus* transcript regulatory network analysis tool (SATRAT).

For any gene that is queried, SATMD generates a list of regulators (consisting of both genes and environmental factors) where the query gene is upregulated or downregulated at the transcript level (Fig. 2). The regulators from this hit list are then used to further deep search SATMD to retrieve an “all/any” and “up/down” relationship among the regulators. The resulting query transcript’s regulatory network data are coded in the JavaScript Object Notation format and displayed as a network (Fig. 3).

The resulting network is interactive, and users have the ability to click, drag, zoom, and pan the network to better understand the regulatory network. Source and targets are clearly marked using arrowheads. Regulatory genes and environmental factors are colored differently to enable easy visualization. SATRAT contains three components: 1. the query results data from the SATMD; 2. a custom PHP script that generates the network based on the query results data; and 3. an open source library—Cytoscape web—for the network visualization (Lopes et al., 2010). Advanced exporting options for the regulatory network will be provided in future versions.

## Results and Discussion

**Figure 4:**
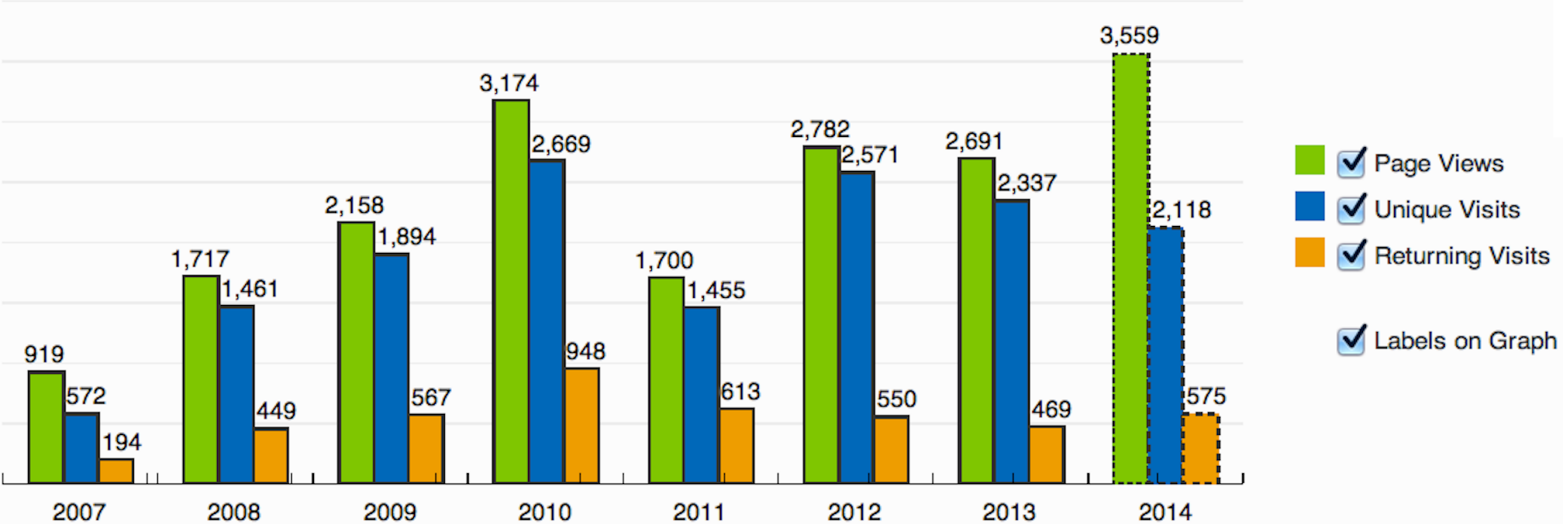
SATMD usage. Distribution of visits to the older version of SATMD.

The first version of this database (SAMMD) is used extensively by numerous researchers throughout the world. Based on the StatCounter, a website visitor analysis tool, this database (as of November 10th, 2014) has had 14,837 unique visits and 4,317 returning visitors from 28 countries that had at least 20 visits. A breakdown of these visits on a yearly basis is given in Fig. 4. Most research institutes have accessed this resource for their research.

The advanced version of this database, SATMD, is designed to include transcriptome data generated through other technologies in the future, and it contains all published data sets in *S. aureus*. It is expected to be a powerful resource for researchers in *S. aureus* and bioinformatics. Because SATMD is open sourced under a GNU-general public license (GPL), similar resources could be built for other organisms. The novel *S. aureus* regulatory network analysis tool (SATRAT), which is based on SATMD, would allow researchers to analyze, understand, and discover hidden regulatory mechanisms and complex interplay between different regulatory elements (Delgado et al., 2008; Cuaron et al., 2013).

### Functional use case of SATRAT

**Figure 5:**
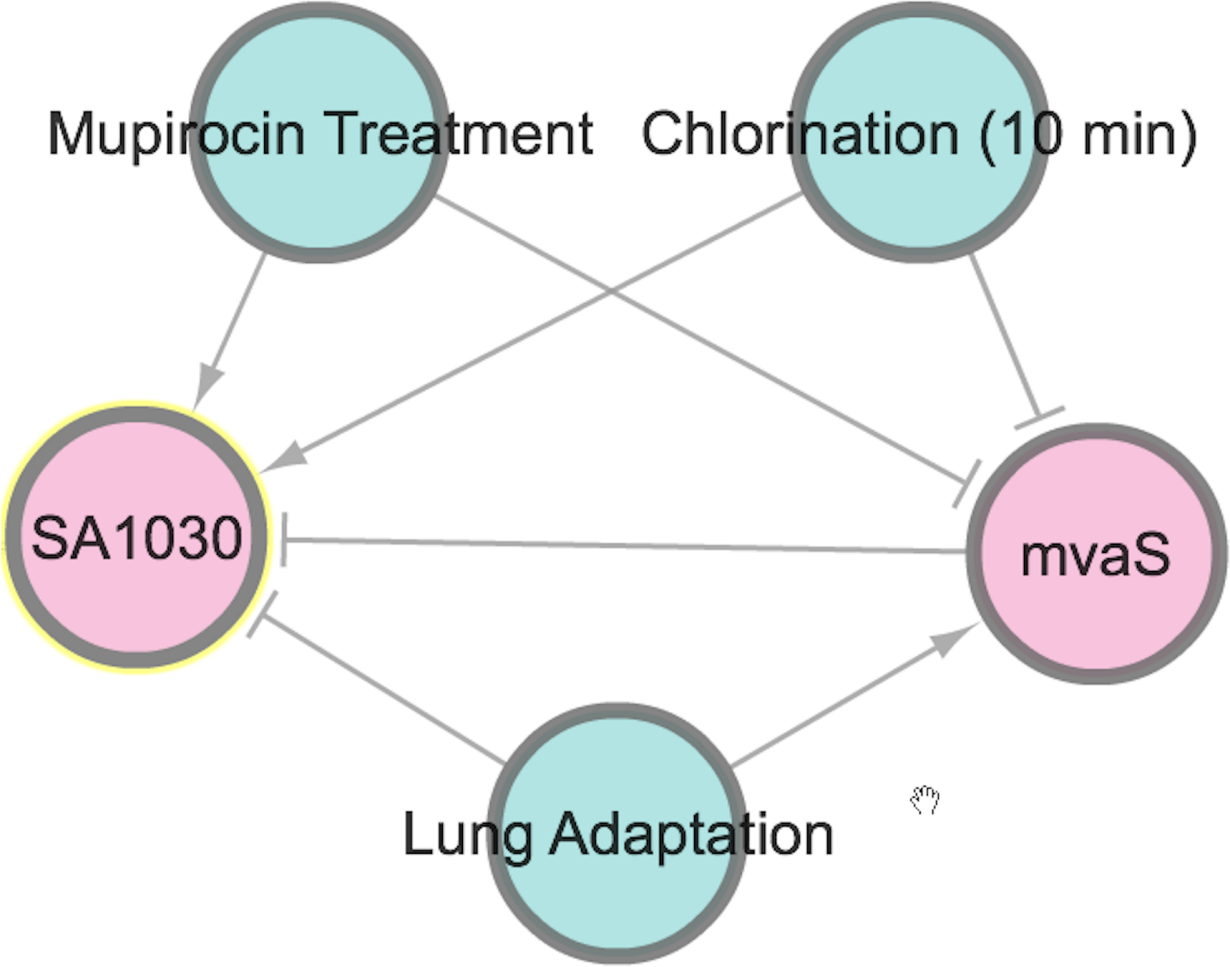
SATRAT case study. Transcript regulatory network of SA1030.

To illustrate the potential use of SATRAT, we used SATMD to examine the expression status of SA1030, a hypothetical protein. This query showed that, apart from other gene regulators, SA1030 is upregulated in “Mupirocin Treatment” and “chlorination” conditions (Fig. 2). It also showed that SA1030 is downregulated by *mvaS* either directly or indirectly. Further analysis of the results shows the associations between the regulators of SA1030, where *mvaS* was downregulated in both the “Mupirocin Treatment” and “chlorination” conditions (Fig. 3). Based on the transcript regulatory network generated (Fig. 5), we hypothesized that SA1030 was potentially regulated under the “Mupirocin Treatment” and “chlorination” conditions through *mvaS*. The same *mvaS*-mediated regulation of SA1030 is also strongly suggested in the “lung adaptation” condition (negative regulatory effect).

Exploring, understanding and discovering relationships (like the one described here between SA1030 and *mvaS*) is now possible using SATRAT. While SATRAT could be an excellent tool to understand *S. aureus* biology, we urge researchers to exercise caution while interpreting results from it, as the data in SATMD comes from a wide variety of experimental conditions, strains and laboratory procedures.

SATMD and SATRAT are licensed under open source GNU-GPL. The website is available at www.bioinformatics.org/sammd/. Most data are readily downloadable through the website. The full data dump and the associated code are available free of cost, upon request. The application has been tested in modern browsers (Firefox, Safari, and Internet Explorer). JavaScript and Flash must be enabled in the browser to be able to use all features of SATMD.

## References

[ref-1] Chambers HF (2001). The changing epidemiology of *Staphylococcus aureus*?. Emerging Infectious Diseases.

[ref-2] Cuaron JA, Dulal S, Song Y, Singh AK, Montelongo CE, Yu W, Nagarajan V, Jayaswal RK, Wilkinson BJ, Gustafson JE (2013). Tea tree oil-induced transcriptional alterations in *Staphylococcus aureus*. Phytotherapy Research.

[ref-3] Delgado A, Zaman S, Muthaiyan A, Nagarajan V, Elasri MO, Wilkinson BJ, Gustafson JE (2008). The fusidic acid stimulon of *Staphylococcus aureus*. Journal of Antimicrobial Chemotherapy.

[ref-4] Diep BA, Otto M (2008). The role of virulence determinants in community-associated MRSA pathogenesis. Trends in Microbiology.

[ref-5] Francis JS, Doherty MC, Lopatin U, Johnston CP, Sinha G, Ross T, Cai M, Hansel NN, Perl T, Ticehurst JR, Carroll K, Thomas DL, Nuermberger E, Bartlett JG (2005). Severe community-onset pneumonia in healthy adults caused by methicillin-resistant *Staphylococcus aureus* carrying the Panton-Valentine leukocidin genes. Clinical Infectious Diseases.

[ref-6] Hidron AI, Low CE, Honig EG, Blumberg HM (2009). Emergence of community-acquired meticillin-resistant *Staphylococcus aureus* strain USA300 as a cause of necrotising community-onset pneumonia. The Lancet Infectious Diseases.

[ref-7] Ibarra JA, Perez-Rueda E, Carroll RK, Shaw LN (2013). Global analysis of transcriptional regulators in *Staphylococcus aureus*. BMC Genomics.

[ref-8] Klevens RM, Morrison MA, Fridkin SK, Reingold A, Petit S, Gershman K, Ray S, Harrison LH, Lynfield R, Dumyati G, Townes JM, Craig AS, Fosheim G, McDougal LK, Tenover FC (2006). Community-associated methicillin-resistant *Staphylococcus aureus* and healthcare risk factors. Emerging Infectious Diseases.

[ref-9] Lopes CT, Franz M, Kazi F, Donaldson SL, Morris Q, Bader GD (2010). Cytoscape web: an interactive web-based network browser. Bioinformatics.

[ref-10] Miller LG, Perdreau-Remington F, Rieg G, Mehdi S, Perlroth J, Bayer AS, Tang AW, Phung TO, Spellberg B (2005). Necrotizing fasciitis caused by community-associated methicillin-resistant *Staphylococcus aureus* in Los Angeles. New England Journal of Medicine.

[ref-11] Nagarajan V, Elasri MO (2007). SAMMD *Staphylococcus aureus* microarray meta-database. BMC Genomics.

[ref-12] Nagarajan V, Smeltzer MS, Elasri MO (2009). Genome-scale transcriptional profiling in *Staphylococcus aureus*: bringing order out of chaos. FEMS Microbiology Letters.

